# A Few Remarks Pertaining to Diagnosis and Treatment of Ordinary Ear Affections

**Published:** 1896-11

**Authors:** F. Pierce Hoover

**Affiliations:** Lecturer in Department of Otology, New York Polyclinic; Assistant Surgeon Manhattan Eye and Ear Hospital, New York City


### A FEW REMARKS PERTAINING TO DIAGNOSIS AND TREATMENT OF ORDINARY EAR AFFECTIONS.

BY F. PIERCE HOOVER, M.D.,

Lecturer in Department of Otology, New York Polyclinic; Assistant Surgeon Manhattan Eye and Ear Hospital, New York City.

It is not my intention to go into the details of the diagnosis and treatment of *all* affections of the ear, but in as few words as possible to suggest a few points which I trust will be of use to some one.

One of the most common complaints is deafness, with or without pain, noises of one kind or another from an accumulation of wax in the ear. To cleanse it out seems simple enough, but requires careful manipulation, and one must be sure *wax* is present; otherwise the drum membrane will likely be ruptured by the pressure of water syringed against it. Never use cold water in the ear; oily substances avail little but are commonly prescribed.

The instruments *all* physicians should have for ordinary every-day use are a head mirror to reflect rays of light into the ear through a speculum; it can be used by putting it on the head with a band or with a handle to hold it; a nest of three specula, either made of rubber or metal as you prefer; the former break more easily than the latter but have the advantage of not being tarnished by acids touching them; a small cotton applicator and pair of forceps; and lastly a syringe; the best is of metal with a long nozzle, which enables us to wash the entire external canal, which a blunt nozzle syringe will not do usually.

*Chronic Catarrh of the Middle Ear, Otitis Media, Catarrhalis Chronica.*—Patients complain of noises in ears which they have had for a longer or shorter time, sometimes temporarily stopping, but usually continuous; also they may complain of deafness, and occasionally they will say they feel at times dizzy. Should you, after diagnosing the case, be asked if your patient can be cured, it would be the best answer to say, see a specialist. These troubles may last for years; sometimes they are incurable and there is very little satisfaction in treating them. Inflation should be tried, either by holding the nose tight and shutting the mouth tight and blowing (Valsalva method), or using the Politzer bag. Should there be a nasopharyngitis or any nose or throat trouble, attention should be given the same at once.

*Acute Catarrh of the Middle Ear, Otitis Media, Catarrhalis Acuta.*—Patient complains of severe pain in the ear. In many cases of old-fashioned "earache," upon looking in the ear a boil (furuncle) will be found causing the mischief, or there may be granulatious or polypi in the ear, or if one is accustomed to examining ears, a puffed out spot on the drum membrane may be noticed, and if so, this is an indication of pus being back of the same and pressing against the drum, causing pain. As soon as a small opening is made, viz.: paracentesis, by the doctor, with a fine small knife-blade, or the membrane ruptures itself, then relief comes frequently instantaneously. If you are afraid to use the knife and wish to hurry the membrane to free itself of pus, order quite warm water syringed in the ear and have a hot water bag or a linen bag filled with hot salt kept in contact with the ear all the time. This will not only feel most comfortable to the patient, but will ease the pain and many times induce sleep. Of course a sedative should be given if required.

Acute suppurative otitis may be the result of an acute catarrhal trouble. The ear discharges pus. Always after cleansing out the canal have the ear inflated so as to force out any pus that may be posterior to the drum membrane. Wipe out this, then with a pledget of cotton on an applicator dipped in a 40-grain nitrate silver to 1-ounce water-solution, touch the ruptured spot on drum. This treatment, if possible, should be done two or three times a week, and the patient should wash the ear out three times a day with warm water and use peroxide of hydrogen after doing so. *Never*, in these acute cases, use boric acid, either in solution or powdered form. Eczema of the ear is frequently the result of irritation, by scratching the ear with a pin, fingernail or hairpin, and sometimes is the result of a running ear, blood disease, etc.

Local applications two or three times per week of tincture of iodine direct to the affected part, or the use of oxide zinc ointment, I prefer, or touching the same with nitrate of silver, or dusting with borax powder. Keep as dry as possible (not using water to the ear) and the eczema will soon get well. Chronic suppurative otitis media is in my experience more often met with than any of the aforementioned diseases, and, as a rule, very good results are obtained in a comparatively short space of time; that is, if the patient can come for treatment *three* times a week, and will diligently persevere in carrying out instructions at home. After washing out ear look for the cause of the discharge; should granulations be seen scrape them out with a small wire curette made expressly for the purpose. Sometimes these granulations are *behind* the drum; possibly polypi may be seen, and if small can be scraped away with the curette, or pulled away, if large, by a pair of forceps. Best use a 4-per-cent. solution of cocaine in the ear to alleviate the pain before operating. The after-treatment is nitrate of silver, which I use in strength from a 40-grain to 1-ounce solution upwards. Boric acid can be used three times daily, or peroxide of hydrogen. I would not advise any one to attempt to operate beyond the drum unless he has done considerable ear work.

Mastoid trouble should be referred to a specialist or a surgeon to be operated on, and where there is swelling back of the ear, marked tenderness upon pressure, and pain, the sooner the knife is used the better.

Ménière's disease and diseases of labyrinth I will not dwell upon; they should at once be sent to a specialist.

Before closing let me add a few words more. Pain in the ear is not always from the ear, but may be the result of either decayed teeth or neuralgia. *Always* examine the former when seeing a patient for the first time who complains of severe pains in the ear; sometimes the removal or filling of a tooth will cure the complaint.

*143 West 45th Street, New York City.*

